# Supplementary material for: Unraveling Therapeutic Opportunities and the Diagnostic Potential of microRNAs for Human Lung Cancer
Source: Pharmaceutics. 2023 Jul 31;15(8):2061. doi: 10.3390/pharmaceutics15082061 (PMC10459057; doi:10.3390/pharmaceutics15082061)
Supplement: Supplementary file 1 [file pharmaceutics-15-02061-s001.zip › pharmaceutics-2481168- Supplementary material.pdf]

pharmaceutics

MDPI

Supplementary materials

Unraveling Therapeutic Opportunities and the Diagnostic Potential of microRNAs for Human Lung Cancer

Osama Sweef [1,2,*], Elsayed Zaabout [3], Ahmed Bakheet [1], Mohamed Halawa [4], Ibrahim Gad [5], Mohamed Akela [6], Ehab Tousson [2], Ashraf Abdelghany [7] and Saori Furuta [1,*]

Table S1. MiRNAs implicated in lung carcinogenesis with their target genes.

miRNA	Function	Target Genes	References
miR-15a	regulates proliferation and apoptosis	Bcl-2, CCND1, Cyclin E1, VEGF	[1,2]
miR-16	regulates proliferation and apoptosis	Bcl-2, CCND1, Cyclin E1, VEGF	[3,4]
miR-17-5p	Promotes proliferation and inhibits apoptosis	PTEN, BIM, E2F1, Cyclin D1, TP53INP1, LKB1/STK11, TIMP2, ZEB1	[5-10]
miR-21	promotes proliferation, invasion, and metastasis	PTEN, PDCD4, TIMP3, RECK	[11,12]
miR-23a	regulates apoptosis and cell cycle	Bcl-2, PTEN, Cyclin D1, CDK6, TET1, GLS2, ST7	[13-18]
miR-34a	Induces apoptosis and inhibits metastasis	Bcl-2, SIRT1, MET, E2F3	[19,20]
miR-92a	promotes proliferation and angiogenesis	PTEN, TIMP3, FZD4, SFRP1	[21,22]
miR-96	promotes proliferation and inhibits apoptosis	FOXO1, KRAS, Bcl-2, Cyclin D1	[23,24]
miR-106a	Promotes proliferation and inhibits apoptosis	PTEN, BIM, E2F1, Cyclin D1	[25,26]
miR-126	regulates angiogenesis and tumor progression	PIK3R2, KRAS, EGFL7, SPRED1	[27-29]
miR-133b	inhibits proliferation and invasion	FSCN1, ROCK1, EGFR, VEGF	[30,31]
miR-135b	Promotes proliferation and migration	APC, CDKN1B, Cyclin D1, JAK2	[32,33]
miR-146a	can regulate NF-κB, the Wnt/β-catenin, and the EGFR pathway	EGFR, ROCK1, Numb, Notch1, IRAK1	[34-38]
miR-155	enhances tumor growth and metastasis	TP53INP1, SOCS1, SHIP1, FOXO3a, E2F2	[39-44]
miR-182	regulating cell proliferation, apoptosis, migration	FOXO1, KLF4, MTSS1, HIF1α	[45-48]
miR-183	Promotes proliferation and migration	EGR1, RASA1, ITGB1, EZH2, E2F3, PTEN, FOXO1, MTDH	[49-52]
miR-184	tumor suppressor by regulating proliferation, apoptosis, migration and invasion	c-Myc, EZH2, SOX9, Wnt7b	[53-57]
miR-195	regulate several key cellular	Bcl-2, Cyclin D1, IRS1, VEGF,	[58-62]

	processes, including cell proliferation, apoptosis, migration, and invasion	Akt	
miR-200	regulate cell proliferation, apoptosis, and epithelial-mesenchymal transition (EMT)	ZEB1, ZEB2, SIRT1, BMI1, TGFBR2 GATA3,	[63-66]
miR-196a2	significantly elevated the risk of lung cancer	HOXC8, ANXA1, ETS1,BCL2	[67-69]
miR-4782 -3p	associated with tumor progression and metastasis	MARCH5, TRIM44, NDUFA4, SLC2A1.	[70-72]
miR-210	regulate various cellular processes, such as cell proliferation, apoptosis, and angiogenesis.	EFNA3, TP53INP1, RAD52	[73-75]
miR-452-5p	Promote cellular proliferation, migration and invasion of lung cancer	IGF2BP1, MMP9, SIRT1	[76-78]
miR-423	Promote the proliferation and invasion of lung cancer	GRHL2, FOXO3, CASTOR1	[79-81]
miR-486	inhibits cell proliferation, invasion and migration	TENM1, PIK3R1, FOXM1TGFBR1, ZBTB7A	[82-84]
miR-608	act as a tumor suppressor by targeting various oncogenes and signaling pathways.	ZEB1, TP63 , MCL1, TGFBR2	[85-89]
miR-32	promote lung cancer cell proliferation, migration, and invasion	PTEN, SLC45A3, E2F2, TP53, FOXO1, KLF4	[90-93]
miR-27a	tumor Suppressor and regulates non-small cell Lung cancer cell proliferation	HOXB8 – PMC, CDKN1B, PTEN, ZBTB10, SPRY2	[94-96]
miR-196a	regulation of apoptosis and inflammation,	HOXA5, ANXA1, CDK6, FOXO1	[97-99]
miR-545	suppresses cell proliferation by directly targeting cyclin D1 and CDK4 genes in lung cancer cell lines	cyclin D1, CDK4, TP53INP1, ZEB2	[100-103]
miR-193a-3p	inhibit cell proliferation, migration, and invasion of lung cancer	SRSF2, HMGB1, RPS6KA3, MCL1	[104-107]
miR-31	tumor suppressive and oncogenic functions in lung cancer	LATS2, SATB2, ITGA5 , FLOT1	[108-110]
miR-124	promotes lung cancer cell proliferation, migration, and invasion	EZH2, ROCK1, CDK4	[111-113]
miR-125a-5p	regulate cell proliferation, migration and apoptosis	ERBB2/HER2, LIN28B, Mcl-1	[114-118]
miR-204-5p	inhibits cell proliferation, migration and has anti-oncogene effects	CXCR4, BCL2, FOXP1, HMGA2, USP47,	[119-122]
miR-145	regulates tumor growth, invasion, and metastasis,	c-Myc, FSCN1, MUC1, EGFR	[123-125]

miR-20a	promotes lung cancer cell proliferation and invasion	RUNX3, PTEN, AKT2	[126-128]
miR-25	promotes cell proliferation and inhibits apoptosis in non-small cell lung cancer cells	BTG2F, BXW7, SMAD7	[129-132]
miR-375	act as a tumor suppressor by inhibiting cell proliferation, migration, and invasion	AEG-1, YAP1, JAK2, AURKA	[133,134]
miR-148b	regulation of several genes and pathways that are crucial for cancer progression and metastasis	WISP2, FGF9, VEGFA, PIK3R1	[135-139]
miR-422a	regulate cell growth and cell cycle	KRAS, CDK4, SP1	[140-145]

Table S2. miRNAs involved in lung carcinogenesis upon exposure to arsenic and BaP.

MiRNA	Function	Target genes	Expression level	References
miR-21	Oncogene	PTEN, PDCD4, TPM1, RECK	Upregulated	[146-148]
miR-31	Oncogene	NOTCH1, SPARC, SATB2	Upregulated	[149-151]
miR-34a	Tumor suppressor	Notch1, Bcl-2, c-Myc, Cyclin D1	Downregulated	[152-154]
miR-126	Tumor suppressor	PI3K/AKT, KRAS, EGFL7	Downregulated	[155-157]
miR-143	Tumor suppressor	KRAS, ERK5	Downregulated	[158,159]
miR-145	Tumor suppressor	OCT4, SOX2, KLF4	Downregulated	[160-162]
miR-155	Oncogene	RHOA, SOCS1, TGFBR2	Upregulated	[163-165]
miR-200	Tumor suppressor	ZEB1, ZEB2	Downregulated	[166-168]
miR-205	Tumor suppressor	ZEB1, ZEB2	Downregulated	[169-171]
miR-214	Oncogene	PTEN, PDCD4, TPM1	Upregulated	[172-174]

Table S3. The signaling pathways in lung carcinogenesis are subject to regulation by miRNAs.

Signaling Pathway	Involved miRNAs	Mechanism	References
Wnt	miR-34a, miR-122, miR-135a/b, miR-148a/b, miR-200c, miR-375	Regulation of cell proliferation, differentiation, and apoptosis	[175-180]
TGF-β	miR-21, miR-29, miR-34, miR-122, miR-155, miR-200, miR-335	Regulation of cell differentiation, migration, and apoptosis	[181-183]
Notch	miR-34, miR-122, miR-150, miR-200, miR-326, miR-449, miR-708	Regulation of cell fate determination, differentiation, and proliferation	[184-188]
Hedgehog	miR-125b, miR-324-5p, miR-326, miR-330-3p, miR-494, miR-615-3p, miR-744	Regulation of cell proliferation, differentiation, and survival	[189-191]
PI3K/Akt	miR-7, miR-21, miR-34, miR-126, miR-143, miR-145, miR-155, miR-221/222, miR-302, miR-375	Regulation of cell growth, survival, and metabolism	[192-198]
MAPK/ERK	miR-21, miR-34, miR-143, miR-146a, miR-150, miR-155, miR-221/222, miR-424, miR-503	Regulation of cell proliferation, differentiation, and survival	[199-207]
JAK/STAT	miR-21, miR-23a, miR-29, miR-30, miR-126, miR-155, miR-223, miR-451	Regulation of cell growth, differentiation, and immune response	[208-215]
NF-κB	miR-9, miR-21, miR-34, miR-146, miR-155, miR-221/222, miR-223, miR-301, miR-365	Regulation of inflammation, cell survival, and immune response	[216-224]
Hippo	miR-125b, miR-200c, miR-429, miR-21, miR-34a	Regulation of Hippo pathway genes, cell proliferation, and apoptosis	[225-229]
Tp53	miR-34a, miR-125b, miR-192, miR-145, miR-155	Regulation of Tp53 and Tp53 target genes, cell cycle arrest, apoptosis, senescence	[230-234]
