# Supplementary material for: Unraveling Therapeutic Opportunities and the Diagnostic Potential of microRNAs for Human Lung Cancer
Source: Pharmaceutics. 2023 Jul 31;15(8):2061. doi: 10.3390/pharmaceutics15082061 (PMC10459057; doi:10.3390/pharmaceutics15082061)
Supplement: Supplementary file 1 [file pharmaceutics-15-02061-s001.zip › Supplementary References of Tables S1-S3.pdf]

References of Supplementary Tables (1-3):

1- Evans, C.D.; LaDow, K.; Schumann, B.L.; Savage, R.E. Jr.; Caruso, J.; Vonderheide, A.; Succop, P.; Talaska, G. Effect of Arsenic on Benzo[a]pyrene DNA Adduct Levels in Mouse Skin and Lung. Carcinogenesis 2004, 25, 493-497. https://doi.org/10.1093/carcin/bgg199.

2- Liu, X.; Xiao, J.; Zhu, H.; Wei, X.; Platt, C.; Damilano, F.; Xiao, C.; Bezzerides, V.; Bostrom, P.; Che, L.; et al. MicroRNA-15a/b Are Up-Regulated in Response to Myocardial Ischemia/Reperfusion Injury. J. Geriatr. Cardiol. 2014, 11, 53-57. Available online: http://www.jgc301.com/en/article/doi/10.3724/SP.J.1263.2012.00028. (accessed on May 2023).

3- Bandres, E.; Cubedo, E.; Agirre, X.; Malumbres, R.; Zárate, R.; Ramirez, N.; Abajo, A.; Navarro, A.; Moreno, I.; Monzó, M.; et al. Identification by Real-Time PCR of 13 Mature MicroRNAs Differentially Expressed in Colorectal Cancer and Non-Tumoral Tissues. Mol. Cancer 2006, 5, 29. https://doi.org/10.1186/1476-4598-5-29.

4- Kovalchuk, O.; Filkowski, J.; Meservy, J.; Ilnytskyy, Y.; Tryndyak, V.P.; Chekhun, V.F.; Pogribny, I.P. Involvement of MicroRNA-451 in Resistance of the MCF-7 Breast Cancer Cells to Chemotherapeutic Drug Doxorubicin. Mol. Cancer Ther. 2008, 7, 2152-2159. https://doi.org/10.1158/1535-7163.MCT-08-0021.

5- Wu, C.; Cao, Y.; He, Z.; He, J.; Hu, C.; Duan, H.; Jiang, J.; Xia, L. MiR-17-5p Promotes Proliferation by Targeting SOCS6 in Non-Small Cell Lung Cancer Cells. FEBS Lett. 2011, 585, 903-908. **https://doi.org/10.1016/j.febslet.2014.04.036**.

6- Wu, Q.; Luo, G.; Yang, Z.; Zhu, F.; An, Y.; Shi, Y.; Fan, D. miR-17-5p Promotes Proliferation by Targeting SOCS6 in Gastric Cancer Cells. FEBS Lett. 2014, 588, 2055-2062. https://doi.org/10.1016/j.febslet.2014.04.036.

7- Liu, X.H.; Lu, K.H.; Wang, K.M.; Sun, M.; Zhang, E.B.; Yang, J.S.; Yin, D.D.; Liu, Z.L.; Zhou, J.; Liu, Z.J.; De, W.; Wang, Z.X. MicroRNA-196a Promotes Non-Small Cell Lung Cancer Cell Proliferation and Invasion through Targeting HOXA5. BMC Cancer 2012, 12, 348. https://doi.org/10.1186/1471-2407-12-348.

8- Xiong M, Wang P, Pan B, Nie J, Wang S, He B. The diagnostic and prognostic values of microRNA-196a in cancer. Biosci Rep. 2021 Jan 29;41(1):BSR20203559. https://doi.org/10.1042/BSR20203559.

9- Shang Y, Wang LQ, Guo QY, Shi TL. MicroRNA-196a overexpression promotes cell proliferation and inhibits cell apoptosis through PTEN/Akt/FOXO1 pathway. Int J Clin Exp Pathol. 2015 Mar 1;8(3):2461-72. Available online: https://pubmed.ncbi.nlm.nih.gov/26045752/. (accessed on May 2023).

10- Larabee SM, Coia H, Jones S, Cheung E, Gallicano GI. miRNA-17 members that target Bmpr2 influence signaling mechanisms important for embryonic stem cell differentiation in vitro and gastrulation in embryos. Stem Cells Dev. 2015 Feb 1;24(3):354-71. https://doi.org/10.1089/scd.2014.0051.

11- Meng F, Henson R, Wehbe-Janek H, Ghoshal K, Jacob ST, Patel T. MicroRNA-21 regulates expression of the PTEN tumor suppressor gene in human hepatocellular cancer. Gastroenterology. 2007 Aug;133(2):647-58. 10.1053/j.gastro.2007.05.022. Epub 2007 May 21. https://doi.org/10.1053/j.gastro.2007.05.022.

12- Zhu S, Wu H, Wu F, Nie D, Sheng S, Mo YY. MicroRNA-21 targets tumor suppressor genes in invasion and metastasis. Cell Res. 2008 Mar;18(3):350-9. Available online: https://www.nature.com/articles/cr200824. (accessed on May 2023).

13- Tsang SM, Oliemuller E, Howard BA. Regulatory roles for SOX11 in development, stem cells and cancer. Semin Cancer Biol. 2020 Dec;67(Pt 1):3-11. https://doi.org/10.1016/j.semcancer.2020.06.015.

14- Gao ZG, Yang P, Huang J, Ding YQ. CircFBXW7 alleviates glioma progression through regulating miR-23a-3p/PTEN axis. Anat Rec (Hoboken). 2021 Feb;304(2):279-290. 10.1002/ar.24410. Epub 2020 Jun 17. Retraction in: Anat Rec (Hoboken). 2021 Dec;304(12):2895. https://doi.org/10.1002/ar.24410.

15- Hu X, Wang Y, Liang H, Fan Q, Zhu R, Cui J, Zhang W, Zen K, Zhang CY, Hou D, Zhou Z, Chen X. miR-23a/b promote tumor growth and suppress apoptosis by targeting PDCD4 in gastric cancer. Cell Death Dis. 2017 Oct 5;8(10):e3059. Available online: https://www.nature.com/articles/cddis2017447. (accessed on May 2023).

16- Frampton AE, Castellano L, Colombo T, Giovannetti E, Krell J, Jacob J, Pellegrino L, Roca-Alonso L, Funel N, Gall TM, Ahmad R, Habib NA, Knösel T, Stebbing J, Jiao LR. Integrated molecular analysis to investigate the role of microRNAs in pancreatic tumour growth and progression. Lancet. 2015 Feb 26;385 Suppl 1:S37. https://doi.org/10.1016/S0140-6736(15)60352-X.

17- Rao Y, Fang Y, Tan W, Liu D, Pang Y, Wu X, Zhang C, Li G. Delivery of Long Non-coding RNA NEAT1 by Peripheral Blood Monouclear Cells-Derived Exosomes Promotes the Occurrence of Rheumatoid Arthritis via the MicroRNA-23a/MDM2/SIRT6 Axis. Front Cell Dev Biol. 2020 Sep 11;8:551681. https://doi.org/10.3389/fcell.2020.551681.

18- Nie M, Yu S, Peng S, Fang Y, Wang H, Yang X. miR-23a and miR-27a promote human granulosa cell apoptosis by targeting SMAD5. Biol Reprod. 2015 Oct;93(4):98. https://doi.org/10.1095/biolreprod.115.130690.

19- Gallardo E, Navarro A, Viñolas N, Marrades RM, Diaz T, Gel B, Quera A, Bandres E, Garcia-Foncillas J, Ramirez J, Monzo M. miR-34a as a prognostic marker of relapse in surgically resected non-small-cell lung cancer. Carcinogenesis. 2009 Nov;30(11):1903-9. https://doi.org/10.1093/carcin/bgp219.

20- Yan X, Tang B, Chen B, Shan Y, Yang H; Reproducibility Project: Cancer Biology. Replication Study: The microRNA miR-34a inhibits prostate cancer stem cells and metastasis by directly repressing CD44. Elife. 2019 Mar 12;8:e43511. https://doi.org/10.7554/eLife.43511.

21- Cai X, Hagedorn CH, Cullen BR. Human microRNAs are processed from capped, polyadenylated transcripts that can also function as mRNAs. RNA. 2004 Dec;10(12):1957-66. Available online: https://rnajournal.cshlp.org/content/10/12/1957. (accessed on May 2023).

22- International BR. Retracted: MicroRNA-124 Regulates the Proliferation of Colorectal Cancer Cells by Targeting iASPP. Biomed Res Int. 2023 Jan 4;2023:9847281. https://doi.org/10.1155/2023/9847281.

23- Hu CB, Li QL, Hu JF, Zhang Q, Xie JP, Deng L. miR-124 inhibits growth and invasion of gastric cancer by targeting ROCK1. Asian Pac J Cancer Prev. 2014;15(16):6543-6. https://doi.org/10.7314/APJCP.2014.15.16.6543.

24- Tang Y, Pan J, Huang S, Peng X, Zou X, Luo Y, Ren D, Zhang X, Li R, He P, Wa Q. Downregulation of miR-133a-3p promotes prostate cancer bone metastasis via activating PI3K/AKT signaling. J Exp Clin Cancer Res. 2018 Jul 18;37(1):160. Available online: https://jeccr.biomedcentral.com/articles/10.1186/s13046-018-0813-4. (accessed on May 2023).

25- Qin Y, Dang X, Li W, Ma Q. miR-133a functions as a tumor suppressor and directly targets FSCN1 in pancreatic cancer. Oncol Res. 2013;21(6):353-63. https://doi.org/10.3727/096504014X14024160459122.

26- Li Z, Zhang W, Huang Y. MiRNA-133a is involved in the regulation of postmenopausal osteoporosis through promoting osteoclast differentiation. Acta Biochim Biophys Sin (Shanghai). 2018 Mar 1;50(3):273-280. https://doi.org/10.1093/abbs/gmy006.

27- Cho, W. C. OncomiRs: The Discovery and Progress of MicroRNAs in Cancers. Mol. Cancer 2007, 6, 60. https://doi.org/10.1186/1476-4598-6-60.

28- Garzon R, Garofalo M, Martelli MP, Briesewitz R, Wang L, Fernandez-Cymering C, Volinia S, Liu CG, Schnittger S, Haferlach T, Liso A, Diverio D, Mancini M, Meloni G, Foa R, Martelli MF, Mecucci C, Croce CM, Falini B. Distinctive microRNA signature of acute myeloid leukemia bearing cytoplasmic mutated nucleophosmin. Proc Natl Acad Sci U S A. 2008 Mar 11;105(10):3945-50. https://doi.org/10.1073/pnas.0800135105.

29- Garzon R, Volinia S, Liu CG, Fernandez-Cymering C, Palumbo T, Pichiorri F, Fabbri M, Coombes K, Alder H, Nakamura T, Flomenberg N, Marcucci G, Calin GA, Kornblau SM, Kantarjian H, Bloomfield CD, Andreeff M, Croce CM. MicroRNA signatures associated with cytogenetics and prognosis in acute myeloid leukemia. Blood. 2008 Mar 15;111(6):3183-9. https://doi.org/10.1182/blood-2007-07-098749.

30- Campayo M, Navarro A, Benítez JC, Santasusagna S, Ferrer C, Monzó M, Cirera L. miR-21, miR-99b and miR-375 combination as predictive response signature for preoperative chemoradiotherapy in rectal cancer. PLoS One. 2018 Nov 2;13(11):e0206542. https://doi.org/10.1371/journal.pone.0206542.

31- Yanaihara N, Caplen N, Bowman E, Seike M, Kumamoto K, Yi M, Stephens RM, Okamoto A, Yokota J, Tanaka T, Calin GA, Liu CG, Croce CM, Harris CC. Unique microRNA molecular profiles in lung cancer diagnosis and prognosis. Cancer Cell. 2006 Mar;9(3):189-98. https://doi.org/10.1016/j.ccr.2006.01.025.

32- Hu, Z.; Chen, X.; Zhao, Y.; Tian, T.; Jin, G.; Shu, Y.; Chen, Y.; Xu, L.; Zen, K.; Zhang, C.; et al. Serum microRNA signatures identified in a genome-wide serum microRNA expression profiling predict survival of non-small-cell lung cancer. J. Clin. Oncol. 2010, 28, 1721-1726. https://doi.org/10.1200/JCO.2009.24.9342.

33- Cui L, Zhou H, Zhao H, Zhou Y, Xu R, Xu X, Zheng L, Xue Z, Xia W, Zhang B, Ding T, Cao Y, Tian Z, Shi Q, He X. MicroRNA-99a induces G1-phase cell cycle arrest and suppresses tumorigenicity in renal cell carcinoma. BMC Cancer. 2012 Nov 23;12:546. 10.1186/1471-2407-12-546. Erratum in: BMC Cancer. 2021 Jan 29;21(1):103. Available online: https://bmccancer.biomedcentral.com/articles/10.1186/1471-2407-12-546. (accessed on May 2023).

34- Li X, Han J, Zhu H, Peng L, Chen Z. miR-181b-5p mediates TGF-β1-induced epithelial-to-mesenchymal transition in non-small cell lung cancer stem-like cells derived from lung adenocarcinoma A549 cells. Int J Oncol. 2017 Jul;51(1):158-168. https://doi.org/10.3892/ijo.2017.4007.

35- Wang G, Huang Y, Wang LL, Zhang YF, Xu J, Zhou Y, Lourenco GF, Zhang B, Wang Y, Ren RJ, Halliday GM, Chen SD. MicroRNA-146a suppresses ROCK1 allowing hyperphosphorylation of tau in Alzheimer's disease. Sci Rep. 2016 May 25;6:26697. Available online: https://www.nature.com/articles/srep26697. (accessed on May 2023).

36- Hu ZJ, He JF, Li KJ, Chen J, Xie XR. Decreased microRNA-146a in CD4+T cells promote ocular inflammation in thyroid-associated ophthalmopathy by targeting NUMB. Eur Rev Med Pharmacol Sci. 2017 Apr;21(8):1803-1809. Available online: https://pubmed.ncbi.nlm.nih.gov/28485799/. (accessed on May 2023).

37- Huang C, Liu XJ, QunZhou, Xie J, Ma TT, Meng XM, Li J. MiR-146a modulates macrophage polarization by inhibiting Notch1 pathway in RAW264.7 macrophages. Int Immunopharmacol. 2016 Mar;32:46-54. https://doi.org/10.1016/j.intimp.2016.01.009.

38- Palomer X, Capdevila-Busquets E, Botteri G, Davidson MM, Rodríguez C, Martínez-González J, Vidal F, Barroso E, Chan TO, Feldman AM, Vázquez-Carrera M. miR-146a targets Fos expression in human cardiac cells. Dis Model Mech. 2015 Sep;8(9):1081-91. https://doi.org/10.1242/dmm.020768.

39- Peng Y, Dong W, Lin TX, Zhong GZ, Liao B, Wang B, Gu P, Huang L, Xie Y, Lu FD, Chen X, Xie WB, He W, Wu SX, Huang J. MicroRNA-155 promotes bladder cancer growth by repressing the tumor suppressor DMTF1. Oncotarget. 2015 Jun 30;6(18):16043-58. 10.18632/oncotarget.3755. https://doi.org/10.18632/oncotarget.3755.

40- Li X, Liu K, Zhou W, Jiang Z. MiR-155 targeting FoxO3a regulates oral cancer cell proliferation, apoptosis, and DDP resistance through targeting FoxO3a. Cancer Biomark. 2020;27(1):105-111. Available online: https://content.iospress.com/articles/cancer-biomarkers/cbm190555. (accessed on May 2023).

41- Li X, Gong Y, Lin X, Lin Q, Luo J, Yu T, Xu J, Chen L, Xu L, Hu Y. Down-regulation of microRNA-155 suppressed Candida albicans induced acute lung injury by activating SOCS1 and inhibiting inflammation response. J Microbiol. 2022 Apr;60(4):402-410. 10.1007/s12275-022-1663-5. Epub 2022 Feb 14. Available online: https://link.springer.com/article/10.1007/s12275-022-1663-5. (accessed on May 2023).

42- Al-Haidari AA, Syk I, Thorlacius H. MiR-155-5p positively regulates CCL17-induced colon cancer cell migration by targeting RhoA. Oncotarget. 2017 Feb 28;8(9):14887-14896. https://doi.org/10.18632/oncotarget.14841.

43- Wu M, Duan Q, Liu X, Zhang P, Fu Y, Zhang Z, Liu L, Cheng J, Jiang H. MiR-155-5p promotes oral cancer progression by targeting chromatin remodeling gene ARID2. Biomed Pharmacother. 2020 Feb;122:109696. https://doi.org/10.1016/j.biopha.2019.109696.

44- Liao WW, Zhang C, Liu FR, Wang WJ. Effects of miR-155 on proliferation and apoptosis by regulating FoxO3a/BIM in liver cancer cell line HCCLM3. Eur Rev Med Pharmacol Sci. 2020 Jul;24(13):7196. Available online: https://www.europeanreview.org/article/21845. (accessed on May 2023).
45- Suzuki R, Amatya VJ, Kushitani K, Kai Y, Kambara T, Takeshima Y. miR-182 and miR-183 Promote Cell Proliferation and Invasion by Targeting FOXO1 in Mesothelioma. Front Oncol. 2018 Oct 22;8:446. https://doi.org/10.3389/fonc.2018.00446.
46- Chang H, Liu YH, Wang LL, Wang J, Zhao ZH, Qu JF, Wang SF. MiR-182 promotes cell proliferation by suppressing FBXW7 and FBXW11 in non-small cell lung cancer. Am J Transl Res. 2018 Apr 15;10(4):1131-1142. Available online: https://www.ncbi.nlm.nih.gov/pmc/articles/PMC5934572/. (accessed on May 2023).
47- Li X, Zhang X, Zhang Q, Lin R. miR-182 contributes to cell proliferation, invasion and tumor growth in colorectal cancer by targeting DAB2IP. Int J Biochem Cell Biol. 2019 Jun;111:27-36. https://doi.org/10.1016/j.biocel.2019.04.002.
48- Wang J, Li J, Shen J, Wang C, Yang L, Zhang X. MicroRNA-182 downregulates metastasis suppressor 1 and contributes to metastasis of hepatocellular carcinoma. BMC Cancer. 2012 Jun 8;12:227. Available online: https://bmccancer.biomedcentral.com/articles/10.1186/1471-2407-12-227. (accessed on May 2023).
49- Sarver AL, Li L, Subramanian S. MicroRNA miR-183 functions as an oncogene by targeting the transcription factor EGR1 and promoting tumor cell migration. Cancer Res. 2010 Dec 1;70(23):9570-80. https://doi.org/10.1158/0008-5472.CAN-10-2074.
50- Fang Z, Tang J, Bai Y, Lin H, You H, Jin H, Lin L, You P, Li J, Dai Z, Liang X, Su Y, Hu Q, Wang F, Zhang ZY. Plasma levels of microRNA-24, microRNA-320a, and microRNA-423-5p are potential biomarkers for colorectal carcinoma. J Exp Clin Cancer Res. 2015 Aug 22;34(1):86. Available online: https://jeccr.biomedcentral.com/articles/10.1186/s13046-015-0198-6. (accessed on May 2023).
51- Lin X, Zheng L, Song H, Xiao J, Pan B, Chen H, Jin X, Yu H. Effects of microRNA-183 on epithelial-mesenchymal transition, proliferation, migration, invasion and apoptosis in human pancreatic cancer SW1900 cells by targeting MTA1. Exp Mol Pathol. 2017 Jun;102(3):522-532. https://doi.org/10.1016/j.yexmp.2017.05.009.
52- Lu YY, Zheng JY, Liu J, Huang CL, Zhang W, Zeng Y. miR-183 induces cell proliferation, migration, and invasion by regulating PDCD4 expression in the SW1990 pancreatic cancer cell line. Biomed Pharmacother. 2015 Mar;70:151-7. 10.1016/j.biopha.2015.01.016. Epub 2015 Jan 13. https://doi.org/10.1016/j.biopha.2015.01.016.
53- Wang YY, Duan SH, Wang GL, Li JL. Integrated mRNA and miRNA expression profile analysis of female and male gonads in Hyriopsis cumingii. Sci Rep. 2021 Jan 12;11(1):665. Available online: https://www.nature.com/articles/s41598-020-80264-7. (accessed on May 2023).
54- Yang D, Feng W, Zhuang Y, Liu J, Feng Z, Xu T, Wang W, Zhu Y, Wang Z. Long non-coding RNA linc00665 inhibits CDKN1C expression by binding to EZH2 and affects cisplatin sensitivity of NSCLC cells. Mol Ther Nucleic Acids. 2021 Jan 20;23:1053-1065. https://doi.org/10.1016/j.omtn.2021.01.013.
55- He J, Ling L, Liu Z, Ren X, Wan L, Tu C, Li Z. Functional interplay between long non-coding RNAs and the Wnt signaling cascade in osteosarcoma. Cancer Cell Int. 2021 Jun 15;21(1):313. 10.1186/s12935-021-02013-8. Available online: https://cancerci.biomedcentral.com/articles/10.1186/s12935-021-02013-8. (accessed on May 2023).
56- Nouri N, Shareghi-Oskoue O, Aghebati-Maleki L, Danaii S, Ahmadian Heris J, Soltani-Zangbar MS, Kamrani A, Yousefi M. Role of miRNAs interference on ovarian functions and premature ovarian failure. Cell Commun Signal. 2022 Dec 23;20(1):198. 10.1186/s12964-022-00992-3. Available online: https://biosignaling.biomedcentral.com/articles/10.1186/s12964-022-00992-3. (accessed on May 2023).
57- Fisher L. Retraction: Long noncoding RNA ANRIL protects cardiomyocytes against hypoxia/reoxygenation injury by sponging miR-195-5p and upregulating Bcl-2. RSC Adv. 2021 Feb 3;11(11):6238. https://doi.org/10.1039/D1RA90058G.

58- Patil N, Allgayer H, Leupold JH. MicroRNAs in the Tumor Microenvironment. Adv Exp Med Biol. 2020;1277:1-31. Available online: https://link.springer.com/chapter/10.1007/978-3-030-50224-9_1. (accessed on May 2023).

59- Nie H, Mu J, Wang J, Li Y. miR-195-5p regulates multi-drug resistance of gastric cancer cells via targeting ZNF139. Oncol Rep. 2018 Sep;40(3):1370-1378. https://doi.org/10.3892/or.2018.6524.

60- Lin X, Wang S, Sun M, Zhang C, Wei C, Yang C, Dou R, Liu Q, Xiong B. miR-195-5p/NOTCH2-mediated EMT modulates IL-4 secretion in colorectal cancer to affect M2-like TAM polarization. J Hematol Oncol. 2019 Feb 26;12(1):20. 10.1186/s13045-019-0708-7. Retraction in: J Hematol Oncol. 2023 Apr 19;16(1):41. Erratum in: J Hematol Oncol. 2019 Nov 22;12(1):122. Available online: https://jhoonline.biomedcentral.com/articles/10.1186/s13045-019-0708-7. (accessed on May 2023).

61- Yin Y, Hong S, Yu S, Huang Y, Chen S, Liu Y, Zhang Q, Li Y, Xiao H. *MiR-195* Inhibits Tumor Growth and Metastasis in Papillary Thyroid Carcinoma Cell Lines by Targeting *CCND1* and *FGF2*. Int J Endocrinol. 2017;2017:6180425. https://doi.org/10.1155/2017/6180425.

62- Cavallari I, Ciccarese F, Sharova E, Urso L, Raimondi V, Silic-Benussi M, D'Agostino DM, Ciminale V. The miR-200 Family of microRNAs: Fine Tuners of Epithelial-Mesenchymal Transition and Circulating Cancer Biomarkers. Cancers (Basel). 2021 Nov 23;13(23):5874. https://doi.org/10.3390/cancers13235874.

63- Wu CL, Ho JY, Chou SC, Yu DS. MiR-429 reverses epithelial-mesenchymal transition by restoring E-cadherin expression in bladder cancer. Oncotarget. 2016 May 3;7(18):26593-603. https://doi.org/10.18632/oncotarget.8557.

64- Yu Y, Kanwar SS, Patel BB, Oh PS, Nautiyal J, Sarkar FH, Majumdar AP. MicroRNA-21 induces stemness by downregulating transforming growth factor beta receptor 2 (TGFβR2) in colon cancer cells. Carcinogenesis. 2012 Jan;33(1):68-76. https://doi.org/10.1093/carcin/bgr246.

65- Tang X, Tu G, Yang G, Wang X, Kang L, Yang L, Zeng H, Wan X, Qiao Y, Cui X, Liu M, Hou Y. Autocrine TGF-β1/miR-200s/miR-221/DNMT3B regulatory loop maintains CAF status to fuel breast cancer cell proliferation. Cancer Lett. 2019 Jun 28;452:79-89. https://doi.org/10.1016/j.canlet.2019.02.044.

66- Yao CX, Wei QX, Zhang YY, Wang WP, Xue LX, Yang F, Zhang SF, Xiong CJ, Li WY, Wei ZR, Zou Y, Zang MX. miR-200b targets GATA-4 during cell growth and differentiation. RNA Biol. 2013 Apr;10(4):465-80. 10.4161/rna.24370. Epub 2013 Apr 1. https://doi.org/10.4161/rna.24370.

67- Qiu H, Xie Z, Tang W, Liu C, Wang Y, Gu H, Zheng Q. Association between microRNA-146a, -499a and -196a-2 SNPs and non-small cell lung cancer: a case-control study involving 2249 subjects. Biosci Rep. 2021 Feb 26;41(2):BSR20201158. https://doi.org/10.1042/BSR20201158.

68- Belvedere R, Saggese P, Pessolano E, Memoli D, Bizzarro V, Rizzo F, Parente L, Weisz A, Petrella A. miR-196a Is Able to Restore the Aggressive Phenotype of Annexin A1 Knock-Out in Pancreatic Cancer Cells by CRISPR/Cas9 Genome Editing. Int J Mol Sci. 2018 Jul 6;19(7):1967. https://doi.org/10.3390/ijms19071967.

69- Mendiola-Soto DK, Bárcenas-López DA, Pérez-Amado CJ, Cruz-Miranda GM, Mejía-Aranguré JM, Ramírez-Bello J, Hidalgo-Miranda A, Jiménez-Morales S. MiRNAs in Hematopoiesis and Acute Lymphoblastic Leukemia. Int J Mol Sci. 2023 Mar 12;24(6):5436. https://doi.org/10.3390/ijms24065436.

70- Choupani J, Nariman-Saleh-Fam Z, Saadatian Z, Ouladsahebmadarek E, Masotti A, Bastami M. Association of mir-196a-2 rs11614913 and mir-149 rs2292832 Polymorphisms With Risk of Cancer: An Updated Meta-Analysis. Front Genet. 2019 Mar 15;10:186. https://doi.org/10.3389/fgene.2019.00186.

71- Szczyrek M, Bitkowska P, Jutrzenka M, Milanowski J. The Role of the Selected miRNAs as Diagnostic, Predictive and Prognostic Markers in Non-Small-Cell Lung Cancer. J Pers Med. 2022 Jul 27;12(8):1227. https://doi.org/10.3390/jpm12081227.

72- Wu N, Zhang C, Bai C, Han YP, Li Q. MiR-4782-3p inhibited non-small cell lung cancer growth via USP14. Cell Physiol Biochem. 2014;33(2):457-67. https://doi.org/10.1159/000358626.

73- Xu G, Liu C, Liang T, Qin Z, Yu CJ, Zhang Z, Jiang J, Chen J, Zhan X. Integrated miRNA-mRNA network revealing the key molecular characteristics of ossification of the posterior longitudinal ligament. Medicine (Baltimore). 2020 May 22;99(21):e20268. Available online: https://journals.lww.com/md-journal/Fulltext/2020/05220/Integrated_miRNA_mRNA_network_revealing_the_key.59.aspx. (accessed on May 2023).

74- Li J, Li Z, Zhao S, Song Y, Si L, Wang X. Identification key genes, key miRNAs and key transcription factors of lung adenocarcinoma. J Thorac Dis. 2020 May;12(5):1917-1933. Available online: https://jtd.amegroups.org/article/view/39577/html. (accessed on May 2023).

75- Tian Y, Li X, Bai C, Yang Z, Zhang L, Luo J. MiR-17-5p promotes the endothelialization of endothelial progenitor cells to facilitate the vascular repair of aneurysm by regulating PTEN-mediated PI3K/AKT/VEGFA pathway. Cell Cycle. 2020 Dec;19(24):3608-3621. https://doi.org/10.1080/15384101.2020.1857958.

76- García-Martínez A, López-Muñoz B, Fajardo C, Cámara R, Lamas C, Silva-Ortega S, Aranda I, Picó A. Increased *E2F1* mRNA and miR-17-5p Expression Is Correlated to Invasiveness and Proliferation of Pituitary Neuroendocrine Tumours. Diagnostics (Basel). 2020 Apr 16;10(4):227. Available online: https://www.mdpi.com/2075-4418/10/4/227. (accessed on May 2023).

77- Bao C, Liu T, Qian L, Xiao C, Zhou X, Ai H, Wang J, Fan W, Pan J. Shikonin inhibits migration and invasion of triple-negative breast cancer cells by suppressing epithelial-mesenchymal transition via miR-17-5p/PTEN/Akt pathway. J Cancer. 2021 Jan 1;12(1):76-88. Available online: https://www.jcancer.org/v12p0076.htm. (accessed on May 2023).

78- Khajehdehi M, Khalaj-Kondori M, Ghasemi T, Jahanghiri B, Damaghi M. Long Noncoding RNAs in Gastrointestinal Cancer: Tumor Suppression Versus Tumor Promotion. Dig Dis Sci. 2021 Feb;66(2):381-397. https://doi.org/10.1007/s10620-020-06200-x.

79- Zhuang J, Fan J, Zhu L, Zhao L, Huang Y, Pan X, Guo T. miR-452-5p suppressed the metastasis of Non-small cell lung cancer through regulating Moesin. J Cancer. 2023 Jul 3;14(11):2015-2022. Available online: https://www.jcancer.org/v14p2015.htm. (accessed on May 2023).

80- Ding B, Ma G, Wang Z, Liang W, Gao W. Mechanisms of Kidney Cell Pyroptosis in Chronic Kidney Disease and the Effects of Traditional Chinese Medicine. Evid Based Complement Alternat Med. 2021 Oct 11;2021:1173324. https://doi.org/10.1155/2021/1173324.

81- Tutarel O, Dangwal S, Bretthauer J, Westhoff-Bleck M, Roentgen P, Anker SD, Bauersachs J, Thum T. Circulating miR-423_5p fails as a biomarker for systemic ventricular function in adults after atrial repair for transposition of the great arteries. Int J Cardiol. 2013 Jul 15;167(1):63-6. https://doi.org/10.1016/j.ijcard.2011.11.082.

82- Zafari N, Bahramy A, Majidi Zolbin M, Emadi Allahyari S, Farazi E, Hassannejad Z, Yekaninejad MS. microRNAs as novel diagnostic biomarkers in endometriosis patients: a systematic review and meta-analysis. Expert Rev Mol Diagn. 2022 Apr;22(4):479-495. https://doi.org/10.1080/14737159.2021.1960508.

83- Liu Z, Zhang L, Toma MA, Li D, Bian X, Pastar I, Tomic-Canic M, Sommar P, Xu Landén N. Integrative small and long RNA omics analysis of human healing and nonhealing wounds discovers cooperating microRNAs as therapeutic targets. Elife. 2022 Aug 12;11:e80322. https://doi.org/10.7554/eLife.80322.

84- Shen X, Li L, Zhang L, Liu W, Wu Y, Ma R. Diagnostic and prognostic value of microRNA-486 in patients with lung cancer: A systematic review and meta-analysis. Int J Biol Markers. 2022 Dec;37(4):377-385. https://doi.org/10.1177/03936155221115.

85- Tian F, Wang J, Ouyang T, Lu N, Lu J, Shen Y, Bai Y, Xie X, Ge Q. MiR-486-5p Serves as a Good Biomarker in Nonsmall Cell Lung Cancer and Suppresses Cell Growth With the Involvement of a Target PIK3R1. Front Genet. 2019 Jul 26;10:688. https://doi.org/10.3389/fgene.2019.00688.

86- Zhang G, Liu Z, Cui G, Wang X, Yang Z. MicroRNA-486-5p targeting PIM-1 suppresses cell proliferation in breast cancer cells. Tumour Biol. 2014 Nov;35(11):11137-45. https://doi.org/10.1007/s13277-014-2412-0.

87- Hsu TK, Asmussen J, Koire A, Choi BK, Gadhikar MA, Huh E, Lin CH, Konecki DM, Kim YW, Pickering CR, Kimmel M, Donehower LA, Frederick MJ, Myers JN, Katsonis P, Lichtarge O. A general calculus of fitness landscapes finds genes under selection in cancers. Genome Res. 2022 May;32(5):916-929. Available online: https://genome.cshlp.org/content/32/5/916. (accessed on May 2023).

88- Li J, Li P, Shao J, Liang S, Wan Y, Zhang Q, Li C, Li Y, Wang C. Emerging Role of Noncoding RNAs in EGFR TKI-Resistant Lung Cancer. Cancers (Basel). 2022 Sep 12;14(18):4423. https://doi.org/10.3390/cancers14184423.

89- Choi YC, Yoon S, Byun Y, Lee G, Kee H, Jeong Y, Yoon J, Baek K. MicroRNA library screening identifies growth-suppressive microRNAs that regulate genes involved in cell cycle progression and apoptosis. Exp Cell Res. 2015 Dec 10;339(2):320-32. https://doi.org/10.1016/j.yexcr.2015.10.012.

90- Wang YF, Ao X, Liu Y, Ding D, Jiao WJ, Yu Z, Zhai WX, Dong SH, He YQ, Guo H, Wang JX. MicroRNA-608 Promotes Apoptosis in Non-Small Cell Lung Cancer Cells Treated With Doxorubicin Through the Inhibition of TFAP4. Front Genet. 2019 Sep 10;10:809. 10.3389/fgene.2019.00809. Erratum in: Front Genet. 2021 Mar 30;12:649586. https://doi.org/10.3389/fgene.2019.00809.

91- Wang Q, He Y, Kan W, Li F, Ji X, Wu X, Wang X, Zhang Y, Chen J. microRNA-32-5p targets KLF2 to promote gastric cancer by activating PI3K/AKT signaling pathway. Am J Transl Res. 2019 Aug 15;11(8):4895-4908. Available online: https://www.ncbi.nlm.nih.gov/pmc/articles/PMC6731418/. (accessed on May 2023).

92- Al-Marzook FA, Hassan DM, Alghazal MW, Kadheem RAA, Jalil AT, Saleh MM. MicroRNA-32 Suppression: its Effects on Prostate Cancer Cells' Capability to Proliferate and Migrate. Drug Res (Stuttg). 2023 Mar;73(3):170-174. online: https://www.thieme-connect.com/products/ejournals/abstract/10.1055/a-1977-8848. (accessed on May 2023).

93- Pacholewska A, Kraft MF, Gerber V, Jagannathan V. Differential Expression of Serum MicroRNAs Supports $CD4^+$ T Cell Differentiation into Th2/Th17 Cells in Severe Equine Asthma. Genes (Basel). 2017 Dec 12;8(12):383. https://doi.org/10.3390/genes8120383.

94- Miao J, Regenstein JM, Xu D, Zhou D, Li H, Zhang H, Li C, Qiu J, Chen X. The roles of microRNA in human cervical cancer. Arch Biochem Biophys. 2020 Sep 15;690:108480. https://doi.org/10.1016/j.abb.2020.108480.

95- Mohamed RH, Pasha HF, Gad DM, Toam MM. miR-146a and miR-196a-2 genes polymorphisms and its circulating levels in lung cancer patients. J Biochem. 2019 May 24:mvz044. https://doi.org/10.1093/jb/mvz044.

96- Yan X, Yu H, Liu Y, Hou J, Yang Q, Zhao Y. miR-27a-3p Functions as a Tumor Suppressor and Regulates Non-Small Cell Lung Cancer Cell Proliferation via Targeting HOXB8. Technol Cancer Res Treat. 2019 Jan 1;18:1533033819861971. https://doi.org/10.1177/1533033819861971.

97- Li S, Han Y, Liang X, Zhao M. LINC01089 inhibits the progression of cervical cancer via inhibiting miR-27a-3p and increasing BTG2. J Gene Med. 2021 Jan;23(1):e3280. https://doi.org/10.1002/jgm.3280.

98- Qiao B, He BX, Cai JH, Tao Q, King-Yin Lam A. MicroRNA-27a-3p Modulates the Wnt/β-Catenin Signaling Pathway to Promote Epithelial-Mesenchymal Transition in Oral Squamous Carcinoma Stem Cells by Targeting SFRP1. Sci Rep. 2017 Apr 20;7:44688. https://doi.org/10.1038/srep44688.

99- Zhao XR, Zhang Z, Gao M, Li L, Sun PY, Xu LN, Qi Y, Yin LH, Peng JY. MicroRNA-27a-3p aggravates renal ischemia/reperfusion injury by promoting oxidative stress via targeting growth factor receptor-bound protein 2. Pharmacol Res. 2020 May;155:104718. https://doi.org/10.1016/j.phrs.2020.104718.

100- Liu T, Qin W, Hou L, Huang Y. MicroRNA-17 promotes normal ovarian cancer cells to cancer stem cells development via suppression of the LKB1-p53-p21/WAF1 pathway. Tumour Biol. 2015 Mar;36(3):1881-93. Available online: https://link.springer.com/article/10.1007/s13277-014-2790-3; (accessed on May 2023).

101- Wang X, Han J, Liu Y, Hu J, Li M, Chen X, Xu L. miR-17-5p and miR-4443 Promote Esophageal Squamous Cell Carcinoma Development by Targeting TIMP2. Front Oncol. 2021 Oct 27;11:605894. https://doi.org/10.3389/fonc.2021.605894.

102- Qu Y, Zhang H, Duan J, Liu R, Deng T, Bai M, Huang D, Li H, Ning T, Zhang L, Wang X, Ge S, Zhou L, Zhong B, Ying G, Ba Y. MiR-17-5p regulates cell proliferation and migration by targeting transforming

growth factor-β receptor 2 in gastric cancer. Oncotarget. 2016 May 31;7(22):33286-96. https://doi.org/10.18632/oncotarget.8946.

103- Du B, Wang Z, Zhang X, Feng S, Wang G, He J, Zhang B. MicroRNA-545 suppresses cell proliferation by targeting cyclin D1 and CDK4 in lung cancer cells. PLoS One. 2014 Feb 5;9(2):e88022. https://doi.org/10.1371/journal.pone.0088022.

104- Zaheer U, Faheem M, Qadri I, Begum N, Yassine HM, Al Thani AA, Mathew S. Expression profile of MicroRNA: An Emerging Hallmark of Cancer. Curr Pharm Des. 2019;25(6):642-653. https://doi.org/10.2174/1386207322666190325122821.

105- Shi X, Ma W, Li Y, Wang H, Pan S, Pan Y, Xu C, Li L. CircPRKCI relieves lipopolysaccharide-induced HK2 cell injury by upregulating the expression of miR-545 target gene ZEB2. Biofactors. 2020 May;46(3):475-486. https://doi.org/10.1002/biof.1620.

106- Deng H, Lv L, Li Y, Zhang C, Meng F, Pu Y, Xiao J, Qian L, Zhao W, Liu Q, Zhang D, Wang Y, Zhang H, He Y, Zhu J. miR-193a-3p regulates the multi-drug resistance of bladder cancer by targeting the LOXL4 gene and the oxidative stress pathway. Mol Cancer. 2014 Oct 14;13:234. https://doi.org/10.1186/1476-4598-13-234.

107- Khoo CP, Roubelakis MG, Schrader JB, Tsaknakis G, Konietzny R, Kessler B, Harris AL, Watt SM. miR-193a-3p interaction with HMGB1 downregulates human endothelial cell proliferation and migration. Sci Rep. 2017 Mar 9;7:44137. https://doi.org/10.1038/srep44137.

108- Jiao Y, Hao L, Xia P, Cheng Y, Song J, Chen X, Wang Z, Ma Z, Zheng S, Chen T, Zhang Y, Yu H. Identification of Potential miRNA-mRNA Regulatory Network Associated with Pig Growth Performance in the Pituitaries of Bama Minipigs and Landrace Pigs. Animals (Basel). 2022 Nov 7;12(21):3058. https://doi.org/10.3390/ani12213058.

109- Lin C, Zhang S, Wang Y, Wang Y, Nice E, Guo C, Zhang E, Yu L, Li M, Liu C, Hu L, Hao J, Qi W, Xu H. Functional Role of a Novel Long Noncoding RNA *TTN-AS1* in Esophageal Squamous Cell Carcinoma Progression and Metastasis. Clin Cancer Res. 2018 Jan 15;24(2):486-498. https://doi.org/10.1158/1078-0432.CCR-17-1851;

110- Xu H, Ma J, Zheng J, Wu J, Qu C, Sun F, Xu S. MiR-31 Functions as a Tumor Suppressor in Lung Adenocarcinoma Mainly by Targeting HuR. Clin Lab. 2016;62(4):711-8. Available online: https://www.clin-lab-publications.com/article/2115. (accessed on May 2023).

111- Hsu HH, Kuo WW, Shih HN, Cheng SF, Yang CK, Chen MC, Tu CC, Viswanadha VP, Liao PH, Huang CY. FOXC1 Regulation of miR-31-5p Confers Oxaliplatin Resistance by Targeting LATS2 in Colorectal Cancer. Cancers (Basel). 2019 Oct 16;11(10):1576. https://doi.org/10.3390/cancers11101576.

112- Zou G, Ji Q, Geng Z, Du X, Jiang L, Liu T. miR-31-5p from placental and peripheral blood exosomes is a potential biomarker to diagnose preeclampsia. Hereditas. 2022 Sep 19;159(1):35. 10.1186/s41065-022-00250-z. Available online: https://hereditasjournal.biomedcentral.com/articles/10.1186/s41065-022-00250-z. (accessed on May 2023).

113- King HW, Michael MZ, Gleadle JM. Hypoxic enhancement of exosome release by breast cancer cells. BMC Cancer. 2012 Sep 24;12:421. https://doi.org/10.1186/1471-2407-12-421.

114- Wang M, Meng B, Liu Y, Yu J, Chen Q, Liu Y. MiR-124 Inhibits Growth and Enhances Radiation-Induced Apoptosis in Non-Small Cell Lung Cancer by Inhibiting STAT3. Cell Physiol Biochem. 2017;44(5):2017-2028. https://doi.org/10.1159/000485907.

115- Sun TY, Li YQ, Zhao FQ, Sun HM, Gao Y, Wu B, Yang S, Ji FQ, Zhou DS. MiR-1-3p and MiR-124-3p Synergistically Damage the Intestinal Barrier in the Ageing Colon. J Crohns Colitis. 2022 May 10;16(4):656-667. https://doi.org/10.1093/ecco-jcc/jjab179.

116- Liu W, Shen S, Tao M, Wang L. Targeting Notch1 inhibits invasion and growth of ovarian cancer cell through regulation of miR-124/flotillin-1 pathway. Int J Clin Exp Pathol. 2017 Jan 1;10(2):1576-84. Available online: https://e-century.us/files/ijcep/10/2/ijcep0043551.pdf. (accessed on May 2023).

117- Shirjang S, Mansoori B, Asghari S, Duijf PHG, Mohammadi A, Gjerstorff M, Baradaran B. MicroRNAs in cancer cell death pathways: Apoptosis and necroptosis. Free Radic Biol Med. 2019 Aug 1;139:1-15. 10.1016/j.freeradbiomed.2019.05.017. Epub 2019 May 15. Erratum in: Free Radic Biol Med. 2020 Jan;146:402. https://doi.org/10.1016/j.freeradbiomed.2019.05.017.

118- Ghoshal-Gupta S, Kutiyanawalla A, Lee BR, Ojha J, Nurani A, Mondal AK, Kolhe R, Rojiani AM, Rojiani MV. TIMP-1 downregulation modulates miR-125a-5p expression and triggers the apoptotic pathway. Oncotarget. 2018 Jan 2;9(10):8941-8956. https://doi.org/10.18632/oncotarget.23832.

119- Hu H, Du L, Nagabayashi G, Seeger RC, Gatti RA. ATM is down-regulated by N-Myc-regulated microRNA-421. Proc Natl Acad Sci U S A. 2010 Jan 26;107(4):1506-11. https://doi.org/10.1073/pnas.090776310.

120- Shen Z, Chai T, Luo F, Liu Z, Xu H, Zhang P, Kang M, Chen S. Loss of miR-204-5p Promotes Tumor Proliferation, Migration, and Invasion Through Targeting YWHAZ/PI3K/AKT Pathway in Esophageal Squamous Cell Carcinoma. Onco Targets Ther. 2020 May 26;13:4679-4690. https://doi.org/10.2147/OTT.S243215.

121- Zhang B, Yin Y, Hu Y, Zhang J, Bian Z, Song M, Hua D, Huang Z. MicroRNA-204-5p inhibits gastric cancer cell proliferation by downregulating USP47 and RAB22A. Med Oncol. 2015 Jan;32(1):331. https://doi.org/10.1007/s12032-014-0331-y.

122- Jia W, Wu Y, Zhang Q, Gao G, Zhang C, Xiang Y. Identification of four serum microRNAs from a genome-wide serum microRNA expression profile as potential non-invasive biomarkers for endometrioid endometrial cancer. Oncol Lett. 2013 Jul;6(1):261-267. https://doi.org/10.3892/ol.2013.1338.

123- Zhang H, Li W, Gu W, Yan Y, Yao X, Zheng J. MALAT1 accelerates the development and progression of renal cell carcinoma by decreasing the expression of miR-203 and promoting the expression of BIRC5. Cell Prolif. 2019 Sep;52(5):e12640. https://doi.org/10.1111/cpr.12640.

124- Liu L, Wang J, Li X, Ma J, Shi C, Zhu H, Xi Q, Zhang J, Zhao X, Gu M. MiR-204-5p suppresses cell proliferation by inhibiting IGFBP5 in papillary thyroid carcinoma. Biochem Biophys Res Commun. 2015 Feb 20;457(4):621-6. https://doi.org/10.1016/j.bbrc.2015.01.037.

125- Feng Y, Zhu J, Ou C, Deng Z, Chen M, Huang W, Li L. MicroRNA-145 inhibits tumour growth and metastasis in colorectal cancer by targeting fascin-1. Br J Cancer. 2014 Apr 29;110(9):2300-9. https://doi.org/10.1038/bjc.2014.122

126- Li B, Ding CM, Li YX, Peng JC, Geng N, Qin WW. MicroRNA-145 inhibits migration and induces apoptosis in human non-small cell lung cancer cells through regulation of the EGFR/PI3K/AKT signaling pathway. Oncol Rep. 2018 Nov;40(5):2944-2954. https://doi.org/10.3892/or.2018.6666.

127- Wu D, Li M, Wang L, Zhou Y, Zhou J, Pan H, Qu P. microRNA-145 inhibits cell proliferation, migration and invasion by targeting matrix metallopeptidase-11 in renal cell carcinoma. Mol Med Rep. 2014 Jul;10(1):393-8. https://doi.org/10.3892/mmr.2014.2149.

128- Zhang Y, Yang X, Wu H, Zhou W, Liu Z. MicroRNA-145 inhibits migration and invasion via inhibition of fascin 1 protein expression in non-small-cell lung cancer cells. Mol Med Rep. 2015 Oct;12(4):6193-8. https://doi.org/10.3892/mmr.2015.4163.

129- Chen Y, Wang X, Cheng J, Wang Z, Jiang T, Hou N, Liu N, Song T, Huang C. MicroRNA-20a-5p targets RUNX3 to regulate proliferation and migration of human hepatocellular cancer cells. Oncol Rep. 2016 Dec;36(6):3379-3386. https://doi.org/10.3892/or.2016.5144.

130- Zhou Q, Dong J, Luo R, Zhou X, Wang J, Chen F. MicroRNA-20a regulates cell proliferation, apoptosis and autophagy by targeting thrombospondin 2 in cervical cancer. Eur J Pharmacol. 2019 Feb 5;844:102-109. https://doi.org/10.1016/j.ejphar.2018.11.043.

131- Huang D, Peng Y, Ma K, Deng X, Tang L, Jing D, Shao Z. MiR-20a, a novel promising biomarker to predict prognosis in human cancer: a meta-analysis. BMC Cancer. 2018 Nov 29;18(1):1189. https://doi.org/10.1186/s12885-018-4907-3.

132- Chen H, Pan H, Qian Y, Zhou W, Liu X. MiR-25-3p promotes the proliferation of triple negative breast cancer by targeting BTG2. Mol Cancer. 2018 Jan 8;17(1):4. https://doi.org/10.1186/s12943-017-0754-0.

133- Xiang J, Hang JB, Che JM, Li HC. MiR-25 is up-regulated in non-small cell lung cancer and promotes cell proliferation and motility by targeting FBXW7. Int J Clin Exp Pathol. 2015 Aug 1;8(8):9147-53. Available online: https://www.ncbi.nlm.nih.gov/pmc/articles/PMC4583891/ . (accessed on May 2023).

134- Sun Y, Guo F, Bagnoli M, Xue FX, Sun BC, Shmulevich I, Mezzanzanica D, Chen KX, Sood AK, Yang D, Zhang W. Key nodes of a microRNA network associated with the integrated mesenchymal subtype of high-grade serous ovarian cancer. Chin J Cancer. 2015 Jan;34(1):28-40. https://doi.org/10.5732/cjc.014.10284.

135- Jayamohan S, Kannan M, Moorthy RK, Rajasekaran N, Jung HS, Shin YK, Arockiam AJV. Dysregulation of miR-375/AEG-1 Axis by Human Papillomavirus 16/18-E6/E7 Promotes Cellular Proliferation, Migration, and Invasion in Cervical Cancer. Front Oncol. 2019 Sep 9;9:847. 10.3389/fonc.2019.00847. Erratum in: Front Oncol. 2021 May 12;11:694442. https://doi.org/10.3389/fonc.2019.00847.

136- Miao L, Liu K, Xie M, Xing Y, Xi T. miR-375 inhibits Helicobacter pylori-induced gastric carcinogenesis by blocking JAK2-STAT3 signaling. Cancer Immunol Immunother. 2014 Jul;63(7):699-711. https://doi.org/10.1007/s00262-014-1550-y.

137- Nishikawa E, Osada H, Okazaki Y, Arima C, Tomida S, Tatematsu Y, Taguchi A, Shimada Y, Yanagisawa K, Yatabe Y, Toyokuni S, Sekido Y, Takahashi T. miR-375 is activated by ASH1 and inhibits YAP1 in a lineage-dependent manner in lung cancer. Cancer Res. 2011 Oct 1;71(19):6165-73. https://doi.org/10.1158/0008-5472.CAN-11-1020.

138- Kahl I, Mense J, Finke C, Boller AL, Lorber C, Győrffy B, Greve B, Götte M, Espinoza-Sánchez NA. The cell cycle-related genes RHAMM, AURKA, TPX2, PLK1, and PLK4 are associated with the poor prognosis of breast cancer patients. J Cell Biochem. 2022 Mar;123(3):581-600. https://doi.org/10.1002/jcb.30205.

139- Hassan N, Zhao JT, Sidhu SB. The role of microRNAs in the pathophysiology of adrenal tumors. Mol Cell Endocrinol. 2017 Nov 15;456:36-43. https://doi.org/10.1016/j.mce.2016.12.011.

140- Katopodis P, Randeva HS, Spandidos DA, Saravi S, Kyrou I, Karteris E. Host cell entry mediators implicated in the cellular tropism of SARS-CoV-2, the pathophysiology of COVID-19 and the identification of microRNAs that can modulate the expression of these mediators (Review). Int J Mol Med. 2022 Feb;49(2):20. https://doi.org/10.3892/ijmm.2021.5075.

141- Zhang H, Ye Q, Du Z, Huang M, Zhang M, Tan H. MiR-148b-3p inhibits renal carcinoma cell growth and pro-angiogenic phenotype of endothelial cell potentially by modulating FGF2. Biomed Pharmacother. 2018 Nov;107:359-367. https://doi.org/10.1016/j.biopha.2018.07.054.

142- Kiełbowski K, Ptaszyński K, Wójcik J, Wojtyś ME. The role of selected non-coding RNAs in the biology of non-small cell lung cancer. Adv Med Sci. 2023 Mar;68(1):121-137. https://doi.org/10.1016/j.advms.2023.02.004.

143- Taefehshokr S, Taefehshokr N, Hemmat N, Hajazimian S, Isazadeh A, Dadebighlu P, Baradaran B. The pivotal role of MicroRNAs in glucose metabolism in cancer. Pathol Res Pract. 2021 Jan;217:153314. https://doi.org/10.1016/j.prp.2020.153314.

144- Li WQ, Zhang JP, Wang YY, Li XZ, Sun L. MicroRNA-422a functions as a tumor suppressor in non-small cell lung cancer through SULF2-mediated TGF-β/SMAD signaling pathway. Cell Cycle. 2019 Aug;18(15):1727-1744. 10.1080/15384101.2019.1632135. Epub 2019 Jun 28. Retraction in: Cell Cycle. 2023 May;22(9):1154. https://doi.org/10.1080/15384101.2019.1632135.

145- Liang H, Wang R, Jin Y, Li J, Zhang S. MiR-422a acts as a tumor suppressor in glioblastoma by targeting PIK3CA. Am J Cancer Res. 2016 Aug 1;6(8):1695-707. PMID: 27648359; PMCID: PMC5004073. Available online: https://www.ncbi.nlm.nih.gov/pmc/articles/PMC5004073/. (accessed on May 2023).

146- Gu J, Zhu X, Li Y, Dong D, Yao J, Lin C, Huang K, Hu H, Fei J. miRNA-21 regulates arsenic-induced anti-leukemia activity in myelogenous cell lines. Med Oncol. 2011 Mar;28(1):211-8. https://doi.org/10.1007/s12032-009-9413-7.

147- Mei Y, Bian C, Li J, Du Z, Zhou H, Yang Z, Zhao RC. miR-21 modulates the ERK-MAPK signaling pathway by regulating SPRY2 expression during human mesenchymal stem cell differentiation. J Cell Biochem. 2013 Jun;114(6):1374-84. https://doi.org/10.1002/jcb.24479.

148- Liu ZL, Wang H, Liu J, Wang ZX. MicroRNA-21 (miR-21) expression promotes growth, metastasis, and chemo- or radioresistance in non-small cell lung cancer cells by targeting PTEN. Mol Cell Biochem. 2013 Jan;372(1-2):35-45. https://doi.org/10.1007/s11010-012-1443-3.

149- Nakagawa Y, Kuranaga Y, Tahara T, Yamashita H, Shibata T, Nagasaka M, Funasaka K, Ohmiya N, Akao Y. Induced miR-31 by 5-fluorouracil exposure contributes to the resistance in colorectal tumors. Cancer Sci. 2019 Aug;110(8):2540-2548. https://doi.org/10.1111/cas.14090.

150- Krejbich P, Birringer M. The Self-Administered Use of Complementary and Alternative Medicine (CAM) Supplements and Antioxidants in Cancer Therapy and the Critical Role of Nrf-2-A Systematic Review. Antioxidants (Basel). 2022 Oct 30;11(11):2149. https://doi.org/10.3390/antiox11112149.

151- Chu KM, Cho CH, Shin VY. Nicotine and gastrointestinal disorders: its role in ulceration and cancer development. Curr Pharm Des. 2013;19(1):5-10. https://doi.org/10.2174/138161213803903038.

152- Sahni M, Bhandari V. Patho-mechanisms of the origins of bronchopulmonary dysplasia. Mol Cell Pediatr. 2021 Dec 11;8(1):21. https://doi.org/10.1186/s40348-021-00129-5.

153- Zhang Y, Ren H, Li J, Xue R, Liu H, Zhu Z, Pan C, Lin Y, Hu A, Gou P, Cai J, Zhou J, Zhu W, Shi X. Elevated HMGB1 expression induced by hepatitis B virus X protein promotes epithelial-mesenchymal transition and angiogenesis through STAT3/miR-34a/NF-κB in primary liver cancer. Am J Cancer Res. 2021 Feb 1;11(2):479-494. Available online: https://www.ncbi.nlm.nih.gov/pmc/articles/PMC7868754/. (accessed on May 2023).

154- Alvanegh AG, Ganji SM, Kamel A, Tavallaie M, Rafati A, Arpanaei A, Dorostkar R, Ghaleh HEG. Comparison of oncolytic virotherapy and nanotherapy as two new miRNA delivery approaches in lung cancer. Biomed Pharmacother. 2021 Aug;140:111755. https://doi.org/10.1016/j.biopha.2021.111755.

155- Xu, Song L, Li D, Gu Y, Wen ZM, Jie J, Zhao D, Peng LP. MicroRNA-126 Targeting PIK3R2 Inhibits NSCLC A549 Cell Proliferation, Migration, and Invasion by Regulation of PTEN/PI3K/AKT Pathway. Clin Lung Cancer. 2016 Sep;17(5):e65-e75. https://doi.org/10.1016/j.cllc.2016.03.012.

156- Chen Q, Chen S, Zhao J, Zhou Y, Xu L. MicroRNA-126: A new and promising player in lung cancer. Oncol Lett. 2021 Jan;21(1):35. https://doi.org/10.3892/ol.2020.12296.

157- Jalil AT, Abdulhadi MA, Al-Ameer LR, Abbas HA, Merza MS, Zabibah RS, Fadhil AA. The emerging role of microRNA-126 as a potential therapeutic target in cancer: a comprehensive review. Pathol Res Pract. 2023 Jun 19;248:154631. https://doi.org/10.1016/j.prp.2023.154631.

158- Wang Y, Li H, Shi Y, Wang S, Xu Y, Li H, Liu D. miR-143-3p impacts on pulmonary inflammatory factors and cell apoptosis in mice with mycoplasmal pneumonia by regulating TLR4/MyD88/NF-κB pathway. Biosci Rep. 2020 Jul 31;40(7):BSR20193419. https://doi.org/10.1042/BSR20193419.

159- Qian X, Yu J, Yin Y, He J, Wang L, Li Q, Zhang LQ, Li CY, Shi ZM, Xu Q, Li W, Lai LH, Liu LZ, Jiang BH. MicroRNA-143 inhibits tumor growth and angiogenesis and sensitizes chemosensitivity to oxaliplatin in colorectal cancers. Cell Cycle. 2013 May 1;12(9):1385-94. https://doi.org/10.4161/cc.24477.

160- Gao W, Yu Y, Cao H, Shen H, Li X, Pan S, Shu Y. Deregulated expression of miR-21, miR-143 and miR-181a in non small cell lung cancer is related to clinicopathologic characteristics or patient prognosis. Biomed Pharmacother. 2010 Jul;64(6):399-408. https://doi.org/10.1016/j.biopha.2010.01.018.

161- Mo D, Yang D, Xiao X, Sun R, Huang L, Xu J. MiRNA-145 suppresses lung adenocarcinoma cell invasion and migration by targeting N-cadherin. Biotechnol Lett. 2017 May;39(5):701-710. 10.1007/s10529-017-2290-9. Epub 2017 Jan 24. https://doi.org/10.1007/s10529-017-2290-9.

162- Hu H, Xu Z, Li C, Xu C, Lei Z, Zhang HT, Zhao J. MiR-145 and miR-203 represses TGF-β-induced epithelial-mesenchymal transition and invasion by inhibiting SMAD3 in non-small cell lung cancer cells. Lung Cancer. 2016 Jul;97:87-94. https://doi.org/10.1016/j.lungcan.2016.04.017.

163- Liu Q, Chen J, Wang B, Zheng Y, Wan Y, Wang Y, Zhou L, Liu S, Li G, Yan Y. Retracted: miR-145 modulates epithelial-mesenchymal transition and invasion by targeting ZEB2 in non-small cell lung cancer cell lines. J Cell Biochem. 2019 May;120(5):8409-8418. https://doi.org/10.1002/jcb.28126.

164- Pottier N, Maurin T, Chevalier B, Puisségur MP, Lebrigand K, Robbe-Sermesant K, Bertero T, Lino Cardenas CL, Courcot E, Rios G, Fourre S, Lo-Guidice JM, Marcet B, Cardinaud B, Barbry P, Mari B. Identification of keratinocyte growth factor as a target of microRNA-155 in lung fibroblasts: implication in epithelial-mesenchymal interactions. PLoS One. 2009 Aug 24;4(8):e6718. https://doi.org/10.1371/journal.pone.0006718.

165- Wang Y, Wang Z, Lu J, Zhang H. Circular RNA circ-PTEN elevates PTEN inhibiting the proliferation of non-small cell lung cancer cells. Hum Cell. 2021 Jul;34(4):1174-1184. https://doi.org/10.1007/s13577-021-00526-y.

166- Zhang H, Zhang C, Feng R, Zhang H, Gao M, Ye L. Investigating the microRNA-mRNA regulatory network in acute myeloid leukemia. Oncol Lett. 2017 Oct;14(4):3981-3988. https://doi.org/10.3892/ol.2017.6686.

167- Zhang D, Cao X, Li J, Zhao G. MiR-210 inhibits NF-κB signaling pathway by targeting DR6 in osteoarthritis. Sci Rep. 2015 Aug 5;5:12775. https://doi.org/10.1038/srep12775.

168- Gee HE, Camps C, Buffa FM, Patiar S, Winter SC, Betts G, Homer J, Corbridge R, Cox G, West CM, Ragoussis J, Harris AL. hsa-mir-210 is a marker of tumor hypoxia and a prognostic factor in head and neck cancer. Cancer. 2010 May 1;116(9):2148-58. https://doi.org/10.1002/cncr.25009.

169- Hisakane K, Seike M, Sugano T, Yoshikawa A, Matsuda K, Takano N, Takahashi S, Noro R, Gemma A. Exosome-derived miR-210 involved in resistance to osimertinib and epithelial-mesenchymal transition in EGFR mutant non-small cell lung cancer cells. Thorac Cancer. 2021 Jun;12(11):1690-1698. https://doi.org/10.1111/1759-7714.13943.

170- Tsuchiya S, Fujiwara T, Sato F, Shimada Y, Tanaka E, Sakai Y, Shimizu K, Tsujimoto G. MicroRNA-210 regulates cancer cell proliferation through targeting fibroblast growth factor receptor-like 1 (FGFRL1). J Biol Chem. 2011 Jan 7;286(1):420-8. https://doi.org/10.1074/jbc.M110.170852.

171- Li Y, Liang C, Ma H, Zhao Q, Lu Y, Xiang Z, Li L, Qin J, Chen Y, Cho WC, Pestell RG, Liang L, Yu Z. miR-221/222 promotes S-phase entry and cellular migration in control of basal-like breast cancer. Molecules. 2014 May 30;19(6):7122-37. https://doi.org/10.3390/molecules19067122.

172- Di Paolo D, Pontis F, Moro M, Centonze G, Bertolini G, Milione M, Mensah M, Segale M, Petraroia I, Borzi C, Suatoni P, Brignole C, Perri P, Ponzoni M, Pastorino U, Sozzi G, Fortunato O. Cotargeting of miR-126-3p and miR-221-3p inhibits PIK3R2 and PTEN, reducing lung cancer growth and metastasis by blocking AKT and CXCR4 signalling. Mol Oncol. 2021 Nov;15(11):2969-2988. https://doi.org/10.1002/1878-0261.13036.

173- Lu X, Yin B, Wang X, Wang F, Li Y, Wang N, Yang X, Jiang W. Long non-coding RNA-ZNF281 upregulates PTEN expression via downregulation of microRNA-221 in non-small cell lung cancer. Oncol Lett. 2020 Sep;20(3):2962-2968. https://doi.org/10.3892/ol.2020.11821.

174- Li S, Chen H, Ren J, Geng Q, Song J, Lee C, Cao C, Zhang J, Xu N. MicroRNA-223 inhibits tissue factor expression in vascular endothelial cells. Atherosclerosis. 2014 Dec;237(2):514-20. 10.1016/j.atherosclerosis.2014.09.033. Epub 2014 Oct 18. https://doi.org/10.1016/j.atherosclerosis.2014.09.033.

175- Si W, Li Y, Shao H, Hu R, Wang W, Zhang K, Yang Q. MiR-34a Inhibits Breast Cancer Proliferation and Progression by Targeting Wnt1 in Wnt/β-Catenin Signaling Pathway. Am J Med Sci. 2016 Aug;352(2):191-9. https://doi.org/10.1016/j.amjms.2016.05.002.

176- Bafico A, Liu G, Yaniv A, Gazit A, Aaronson SA. Novel mechanism of Wnt signalling inhibition mediated by Dickkopf-1 interaction with LRP6/Arrow. Nat Cell Biol. 2001 Jul;3(7):683-6. https://doi.org/10.1038/35083081.

177- Fathi S, Guessous F, Karkouri M. Diagnostic Value of Potential MicroRNAs in CRC: A Meta-Analysis. Microrna. 2022;11(3):190-205. Available online: https://www.eurekaselect.com/article/123847. (accessed on May 2023).

178- Tang Y, Yang P, Zhu Y, Su Y. LncRNA TUG1 contributes to ESCC progression via regulating miR-148a-3p/MCL-1/Wnt/β-catenin axis in vitro. Thorac Cancer. 2020 Jan;11(1):82-94. https://doi.org/10.1111/1759-7714.13236.

179- Hwang WL, Jiang JK, Yang SH, Huang TS, Lan HY, Teng HW, Yang CY, Tsai YP, Lin CH, Wang HW, Yang MH. MicroRNA-146a directs the symmetric division of Snail-dominant colorectal cancer stem cells. Nat Cell Biol. 2014 Mar;16(3):268-80. 10.1038/ncb2910. Erratum in: Nat Cell Biol. 2014 Apr;16(4):383. Erratum in: Nat Cell Biol. 2019 May;21(5):664. Available online: https://www.nature.com/articles/ncb2910. (accessed on May 2023).

180- Bhatnagar N, Li X, Padi SK, Zhang Q, Tang MS, Guo B. Downregulation of miR-205 and miR-31 confers resistance to chemotherapy-induced apoptosis in prostate cancer cells. Cell Death Dis. 2010 Dec 9;1(12):e105 https://doi.org/10.1038/cddis.2010.85.

181- Davis BN, Hilyard AC, Nguyen PH, Lagna G, Hata A. Smad proteins bind a conserved RNA sequence to promote microRNA maturation by Drosha. Mol Cell. 2010 Aug 13;39(3):373-84. https://doi.org/10.1016/j.molcel.2010.07.011.

182- Gong J, Zhang JP, Li B, Zeng C, You K, Chen MX, Yuan Y, Zhuang SM. MicroRNA-125b promotes apoptosis by regulating the expression of Mcl-1, Bcl-w and IL-6R. Oncogene. 2013 Jun 20;32(25):3071-9. https://doi.org/10.1038/onc.2012.318/.

183- Yan S, Wang M, Zhao J, Zhang H, Zhou C, Jin L, Zhang Y, Qiu X, Ma B, Fan Q. MicroRNA-34a affects chondrocyte apoptosis and proliferation by targeting the SIRT1/p53 signaling pathway during the pathogenesis of osteoarthritis. Int J Mol Med. 2016 Jul;38(1):201-9. https://doi.org/10.3892/ijmm.2016.2618.

184- Xiang W, Lin H, Wang Q, Chen W, Liu Z, Chen H, Zhang H, Chen W. miR-34a suppresses proliferation and induces apoptosis of human lens epithelial cells by targeting E2F3. Mol Med Rep. 2016 Dec;14(6):5049-5056. https://doi.org/10.3892/mmr.2016.5901.

185- Rusu MC, Pop F, Hostiuc S, Manta L, Măru N, Grigoriu M. Transdifferentiations and heterogeneity in the stromal niches of uterine leiomyomas. Rom J Morphol Embryol. 2018;59(3):663-672 Available online https://rjme.ro/RJME/resources/files/590318663672.pdf. (accessed on May 2023).

186- Liu J, Chen W, Zhang H, Liu T, Zhao L. miR-214 targets the PTEN-mediated PI3K/Akt signaling pathway and regulates cell proliferation and apoptosis in ovarian cancer. Oncol Lett. 2017 Nov;14(5):5711-5718. https://doi.org/10.3892/ol.2017.6953.

187- Galardi S, Mercatelli N, Giorda E, Massalini S, Frajese GV, Ciafrè SA, Farace MG. miR-221 and miR-222 expression affects the proliferation potential of human prostate carcinoma cell lines by targeting p27Kip1. J Biol Chem. 2007 Aug 10;282(32):23716-24. https://doi.org/10.1074/jbc.M701805200.

188- Zhang DM, Deng JJ, Wu YG, Tang T, Xiong L, Zheng YF, Xu XM. MicroRNA-223-3p Protect Against Radiation-Induced Cardiac Toxicity by Alleviating Myocardial Oxidative Stress and Programmed Cell Death *via* Targeting the AMPK Pathway. Front Cell Dev Biol. 2022 Jan 17;9:801661. https://doi.org/10.3389/fcell.2021.801661.

189- Zhang X, Zhao X, Shao S, Zuo X, Ning Q, Luo M, Gu S, Zhao X. Notch1 induces epithelial-mesenchymal transition and the cancer stem cell phenotype in breast cancer cells and STAT3 plays a key role. Int J Oncol. 2015 Mar;46(3):1141-8. https://doi.org/10.3892/ijo.2014.2809.

190- Guoping M, Ran L, Yanru Q. miR-143 Inhibits Cell Proliferation of Gastric Cancer Cells Through Targeting GATA6. Oncol Res. 2018 Aug 23;26(7):1023-1029. 10.3727/096504018X15151515028670. Epub 2018 Jan 10. https://doi.org/10.3727/096504018X15151515028670.

191- Jiang Z, Cushing L, Ai X, Lü J. miR-326 is downstream of Sonic hedgehog signaling and regulates the expression of Gli2 and smoothened. Am J Respir Cell Mol Biol. 2014 Aug;51(2):273-83. https://doi.org/10.1165/rcmb.2013-0127OC.

192- Xie H, Zhao Q, Yu L, Lu J, Peng K, Xie N, Ni J, Li B. Circular RNA circ_0047744 suppresses the metastasis of pancreatic ductal adenocarcinoma by regulating the miR-21/SOCS5 axis. Biochem Biophys Res Commun. 2022 May 21;605:154-161. https://doi.org/10.1016/j.bbrc.2022.03.082.

193- Yadav V, Sharma K, Bhattacharya S, Talwar P, Purohit PK, Saini N. hsa-miR-23a~27a~24-2 cluster members inhibit aggressiveness of breast cancer cells by commonly targeting NCOA1, NLK and RAP1B. Life Sci. 2022 Oct 15;307:120906. https://doi.org/10.1016/j.lfs.2022.120906.

194- Wang H, Zhu Y, Zhao M, Wu C, Zhang P, Tang L, Zhang H, Chen X, Yang Y, Liu G. miRNA-29c suppresses lung cancer cell adhesion to extracellular matrix and metastasis by targeting integrin β1 and matrix metalloproteinase2 (MMP2). PLoS One. 2013 Aug 6;8(8):e70192. https://doi.org/10.1371/journal.pone.0070192.

195- Chen C, Tang J, Xu S, Zhang W, Jiang H. miR-30a-5p Inhibits Proliferation and Migration of Lung Squamous Cell Carcinoma Cells by Targeting FOXD1. Biomed Res Int. 2020 Apr 13;2020:2547902. https://doi.org/10.1155/2020/2547902.

196- Sun Y, Bai Y, Zhang F, Wang Y, Guo Y, Guo L. miR-126 inhibits non-small cell lung cancer cells proliferation by targeting EGFL7. Biochem Biophys Res Commun. 2010 Jan 15;391(3):1483-9. https://doi.org/10.1016/j.bbrc.2009.12.098.

197- Wang J, Guo J, Fan H. MiR-155 regulates the proliferation and apoptosis of pancreatic cancer cells through targeting SOCS3. Eur Rev Med Pharmacol Sci. 2020 Dec;24(24):12625. Available online https://www.europeanreview.org/article/24143. (accessed on May 2023).

198- Zhao FY, Han J, Chen XW, Wang J, Wang XD, Sun JG, Chen ZT. miR-223 enhances the sensitivity of non-small cell lung cancer cells to erlotinib by targeting the insulin-like growth factor-1 receptor. Int J Mol Med. 2016 Jul;38(1):183-91. https://doi.org/10.3892/ijmm.2016.2588.

199- Meng F, Henson R, Lang M, Wehbe H, Maheshwari S, Mendell JT, Jiang J, Schmittgen TD, Patel T. Involvement of human micro-RNA in growth and response to chemotherapy in human cholangiocarcinoma cell lines. Gastroenterology. 2006 Jun;130(7):2113-29. https://doi.org/10.1053/j.gastro.2006.02.057.

200- Sorel O, Dewals BG. MicroRNAs in large herpesvirus DNA genomes: recent advances. Biomol Concepts. 2016 Aug 1;7(4):229-39. https://doi.org/10.1515/bmc-2016-0017.

201- Chen CZ. MicroRNAs as oncogenes and tumor suppressors. N Engl J Med. 2005 Oct 27;353(17):1768-71. Available online: https://www.nejm.org/doi/full/10.1056/NEJMp058190. (accessed on May 2023).

202- Tan W, Liao Y, Qiu Y, Liu H, Tan D, Wu T, Tang M, Zhang S, Wang H. miRNA 146a promotes chemotherapy resistance in lung cancer cells by targeting DNA damage inducible transcript 3 (CHOP). Cancer Lett. 2018 Aug 1;428:55-68. https://doi.org/10.1016/j.canlet.2018.04.028.

203- Uddin A, Chakraborty S. Role of miRNAs in lung cancer. J Cell Physiol. 2018 Apr 20. https://doi.org/10.1002/jcp.26607.

204- Wei, J., Gao, W., Zhu, C.-J., Liu, Y.-Q., Mei, Z., Cheng, T., Shu, Y.-Q., and Wu, M. (2012). Identification of plasma microRNA-21 as a biomarker for early detection and chemosensitivity of non-small cell lung cancer. Chin. J. Cancer 31, 433–438. https://doi.org/10.5732/cjc.011.10326.

205- Gao W, Lu X, Liu L, Xu J, Feng D, Shu Y. MiRNA-21: a biomarker predictive for platinum-based adjuvant chemotherapy response in patients with non-small cell lung cancer. Cancer Biol Ther. 2012 Mar;13(5):330-40. https://doi.org/10.4161/cbt.19073;

206- Ferragut Cardoso AP, Udoh KT, States JC. Arsenic-induced changes in miRNA expression in cancer and other diseases. Toxicol Appl Pharmacol. 2020 Dec 15;409:115306. https://doi.org/10.1016/j.taap.2020.115306.

207- Hu Z, Chen X, Zhao Y, Tian T, Jin G, Shu Y, Chen Y, Xu L, Zen K, Zhang C, Shen H. Serum microRNA signatures identified in a genome-wide serum microRNA expression profiling predict survival of non-small-cell lung cancer. J Clin Oncol. 2010 Apr 1;28(10):1721-6. Available online: https://ascopubs.org/doi/10.1200/JCO.2009.24.9342. (accessed on May 2023).

208- Meng F, Henson R, Lang M, Wehbe H, Maheshwari S, Mendell JT, Jiang J, Schmittgen TD, Patel T. Involvement of human micro-RNA in growth and response to chemotherapy in human cholangiocarcinoma cell lines. Gastroenterology. 2006 Jun;130(7):2113-29. https://doi.org/10.1053/j.gastro.2006.02.057.

209- Ma F, Li W, Liu C, Li W, Yu H, Lei B, Ren Y, Li Z, Pang D, Qian C. MiR-23a promotes TGF-β1-induced EMT and tumor metastasis in breast cancer cells by directly targeting CDH1 and activating Wnt/β-catenin signaling. Oncotarget. 2017 Jun 9;8(41):69538-69550. https://doi.org/10.18632/oncotarget.18422.

210- Fabbri M, Garzon R, Cimmino A, Liu Z, Zanesi N, Callegari E, Liu S, Alder H, Costinean S, Fernandez-Cymering C, Volinia S, Guler G, Morrison CD, Chan KK, Marcucci G, Calin GA, Huebner K, Croce CM. MicroRNA-29 family reverts aberrant methylation in lung cancer by targeting DNA methyltransferases 3A and 3B. Proc Natl Acad Sci U S A. 2007 Oct 2;104(40):15805-10. https://doi.org/10.1073/pnas.0707628104.

211- Wang X, Qiu H, Tang R, Song H, Pan H, Feng Z, Chen L. miR-30a inhibits epithelial-mesenchymal transition and metastasis in triple-negative breast cancer by targeting ROR1. Oncol Rep. 2018 Jun;39(6):2635-2643. https://doi.org/10.3892/or.2018.6379.

212- Zhu X, Li H, Long L, Hui L, Chen H, Wang X, Shen H, Xu W. miR-126 enhances the sensitivity of non-small cell lung cancer cells to anticancer agents by targeting vascular endothelial growth factor A. Acta Biochim Biophys Sin (Shanghai). 2012 Jun;44(6):519-26. https://doi.org/10.1093/abbs/gms026.

213- Sun X, Guan G, Dai Y, Zhao P, Liu L, Wang Q, Li X. microRNA-155-5p initiates childhood acute lymphoblastic leukemia by regulating the IRF4/CDK6/CBL axis. Lab Invest. 2022 Apr;102(4):411-421. https://doi.org/10.1038/s41374-021-00638-x.

214- Liu Q, Zhang M, Jiang X, Zhang Z, Dai L, Min S, Wu X, He Q, Liu J, Zhang Y, Zhang Z, Yang R. miR-223 suppresses differentiation of tumor-induced $CD11b^+$ $Gr1^+$ myeloid-derived suppressor cells from bone marrow cells. Int J Cancer. 2011 Dec 1;129(11):2662-73. https://doi.org/10.1002/ijc.25921.

215- Wang XC, Tian LL, Jiang XY, Wang YY, Li DG, She Y, Chang JH, Meng AM. The expression and function of miRNA-451 in non-small cell lung cancer. Cancer Lett. 2011 Dec 8;311(2):203-9. https://doi.org/10.1016/j.canlet.2011.07.026.

216- Jang MH, Kim HJ, Gwak JM, Chung YR, Park SY. Prognostic value of microRNA-9 and microRNA-155 expression in triple-negative breast cancer. Hum Pathol. 2017 Oct;68:69-78. https://doi.org/10.1016/j.humpath.2017.08.026.

217- Coppola V, Musumeci M, Patrizii M, Cannistraci A, Addario A, Maugeri-Saccà M, Biffoni M, Francescangeli F, Cordenonsi M, Piccolo S, Memeo L, Pagliuca A, Muto G, Zeuner A, De Maria R, Bonci D. BTG2 loss and miR-21 upregulation contribute to prostate cell transformation by inducing luminal markers expression and epithelial-mesenchymal transition. Oncogene. 2013 Apr 4;32(14):1843-53. Available online: https://www.nature.com/articles/onc2012194. (accessed on May 2023).

218- Yu G, Li H, Wang J, Gumireddy K, Li A, Yao W, Tang K, Xiao W, Hu J, Xiao H, Lang B, Ye Z, Huang Q, Xu H. miRNA-34a suppresses cell proliferation and metastasis by targeting CD44 in human renal carcinoma cells. J Urol. 2014 Oct;192(4):1229-37. https://doi.org/10.1016/j.juro.2014.05.094.

219- Ye EA, Steinle JJ. miR-146a suppresses STAT3/VEGF pathways and reduces apoptosis through IL-6 signaling in primary human retinal microvascular endothelial cells in high glucose conditions. Vision Res. 2017 Oct;139:15-22. https://doi.org/10.1016/j.visres.2017.03.009.

220- Zheng L, Xu CC, Chen WD, Shen WL, Ruan CC, Zhu LM, Zhu DL, Gao PJ. MicroRNA-155 regulates angiotensin II type 1 receptor expression and phenotypic differentiation in vascular adventitial fibroblasts. Biochem Biophys Res Commun. 2010 Oct 1;400(4):483-8. https://doi.org/10.1016/j.bbrc.2010.08.067.

221- Liang YK, Lin HY, Dou XW, Chen M, Wei XL, Zhang YQ, Wu Y, Chen CF, Bai JW, Xiao YS, Qi YZ, Kruyt FAE, Zhang GJ. MiR-221/222 promote epithelial-mesenchymal transition by targeting Notch3 in breast cancer cell lines. NPJ Breast Cancer. 2018 Aug 6;4:20. Available online https://www.nature.com/articles/s41523-018-0073-7. (accessed on May 2023).

222- Wang R, Wang FF, Cao HW, Yang JY. MiR-223 regulates proliferation and apoptosis of IL-22-stimulated HaCat human keratinocyte cell lines via the PTEN/Akt pathway. Life Sci. 2019 Aug 1;230:28-34. https://doi.org/10.1016/j.lfs.2019.05.045.

223- Zhang W, Zhang T, Jin R, Zhao H, Hu J, Feng B, Zang L, Zheng M, Wang M. MicroRNA-301a promotes migration and invasion by targeting TGFBR2 in human colorectal cancer. J Exp Clin Cancer Res. 2014 Dec 31;33(1):113. Available online: https://jeccr.biomedcentral.com/articles/10.1186/s13046-014-0113-6. (accessed on May 2023).

224- Lee SS, Cheah YK. The Interplay between MicroRNAs and Cellular Components of Tumour Microenvironment (TME) on Non-Small-Cell Lung Cancer (NSCLC) Progression. J Immunol Res. 2019 Feb 13;2019:3046379. https://doi.org/10.1155/2019/3046379.

225- Li W, Sun Z, Chen C, Wang L, Geng Z, Tao J. Sirtuin7 has an oncogenic potential via promoting the growth of cholangiocarcinoma cells. Biomed Pharmacother. 2018 Apr;100:257-266. https://doi.org/10.1016/j.biopha.2018.02.007.

226- Zhao GZ, Niu YQ, Li ZM, Kou D, Zhang L. MiR-200c inhibits proliferation and promotes apoptosis of Wilms tumor cells by regulating akt signaling pathway. Eur Rev Med Pharmacol Sci. 2020 Jun;24(12):6623-6631. Available online: https://www.europeanreview.org/article/21648. (accessed on May 2023).

227- Liu F, Li T, Zhan X. Silencing circular RNAPTPN12 promoted the growth of keloid fibroblasts by activating Wnt signaling pathway via targeting microRNA-21-5p. Bioengineered. 2022 Feb;13(2):3503-3515. https://doi.org/10.1080/21655979.2022.2029108.

228- Lv L, Wang X, Ma T. microRNA-944 Inhibits the Malignancy of Hepatocellular Carcinoma by Directly Targeting IGF-1R and Deactivating the PI3K/Akt Signaling Pathway [Retraction]. Cancer Manag Res. 2021 Jun 16;13:4765. https://doi.org/10.2147/CMAR.S324204.

229- Guan N, Wang R, Feng X, Li C, Guo W. Long non-coding RNA NBAT1 inhibits the progression of glioma through the miR-21/SOX7 axis. Oncol Lett. 2020 Sep;20(3):3024-3034. https://doi.org/10.3892/ol.2020.11847.

230- Pan J, Zhou L, Lin C, Xue W, Chen P, Lin J. MicroRNA-34a Promotes Ischemia-Induced Cardiomyocytes Apoptosis through Targeting Notch1. Evid Based Complement Alternat Med. 2022 Feb 28;2022:1388415. https://doi.org/10.1155/2022/1388415.

231- Zheng, Z., Qu, JQ., Yi, HM. et al. MiR-125b regulates proliferation and apoptosis of nasopharyngeal carcinoma by targeting A20/NF-κB signaling pathway. Cell Death Dis 8, e2855 (2017). https://doi.org/10.1038/cddis.2017.211.

232- Liang J, Cao D, Zhang X, Liu L, Tan Q, Shi S, Chen K, Liang J, Wang Z. miR-192-5p suppresses uterine receptivity formation through impeding epithelial transformation during embryo implantation. Theriogenology. 2020 Nov;157:360-371. https://doi.org/10.1016/j.theriogenology.2020.08.009.

233- Pan S, Zhao X, Shao C, Fu B, Huang Y, Zhang N, Dou X, Zhang Z, Qiu Y, Wang R, Jin M, Kong D. STIM1 promotes angiogenesis by reducing exosomal miR-145 in breast cancer MDA-MB-231 cells. Cell Death Dis. 2021 Jan 4;12(1):38. Available online: https://www.nature.com/articles/s41419-020-03304-0. (accessed on May 2023).

234- Li N, Cui T, Guo W, Wang D, Mao L. MiR-155-5p accelerates the metastasis of cervical cancer cell via targeting TP53INP1. Onco Targets Ther. 2019 Apr 29;12:3181-3196. https://doi.org/10.2147/OTT.S193097.
